# Cryptoendolithic Antarctic Black Fungus *Cryomyces antarcticus* Irradiated with Accelerated Helium Ions: Survival and Metabolic Activity, DNA and Ultrastructural Damage

**DOI:** 10.3389/fmicb.2017.02002

**Published:** 2017-10-17

**Authors:** Claudia Pacelli, Laura Selbmann, Ralf Moeller, Laura Zucconi, Akira Fujimori, Silvano Onofri

**Affiliations:** ^1^Department of Ecological and Biological Sciences, University of Tuscia, Viterbo, Italy; ^2^German Aerospace Center, Institute of Aerospace Medicine, Radiation Biology Department, Space Microbiology Research Group, Cologne, Germany; ^3^National Institute of Radiological Sciences, Research Center for Charged Particle Therapy, Chiba, Japan

**Keywords:** cosmic rays, extremophiles, extremotolerance, fungi, HZE particles, He^2+^ ions, space radiation environment

## Abstract

Space represents an extremely harmful environment for life and survival of terrestrial organisms. In the last decades, a considerable deal of attention was paid to characterize the effects of spaceflight relevant radiation on various model organisms. The aim of this study was to test the survival capacity of the cryptoendolithic black fungus *Cryomyces antarcticus* CCFEE 515 to space relevant radiation, to outline its endurance to space conditions. In the frame of an international radiation campaign, dried fungal colonies were irradiated with accelerated Helium ion (150 MeV/n, LET 2.2 keV/μm), up to a final dose of 1,000 Gy, as one of the space-relevant ionizing radiation. Results showed that the fungus maintained high survival and metabolic activity with no detectable DNA and ultrastructural damage, even after the highest dose irradiation. These data give clues on the resistance of life toward space ionizing radiation in general and on the resistance and responses of eukaryotic cells in particular.

## Introduction

In looking for life on other planets, a robust and detailed understanding of the limits of radiation resistance of Earth extremophiles is extremely important. Indeed, beyond the shielding influence of Earth’s magnetic field and atmosphere, strong ionizing radiations pervade the space environment, representing the major hazard for microbial survival, persistence of detectable biosignatures, and operation of spacecraft equipment (Dartnell, 2011). GCR, SEP, and trapped energetic particles in a planetary magnetic field are natural sources of radiation in space. Although only 1% of GCRs is composed by particles of high (H) charge (Z) and high energy (E) (HZE), i.e., He and Fe ions, the HZE particles are considered one of the major damage for microorganisms accidentally traveling in space (Durante and Cucinotta, 2011). Being high-LET (linear energy transfer) radiation, they cause densely packed lesions characterized by locally multiplied damaged types and sites, which are known to be the major causes of the lethal and mutagenic effects of ionizing radiation (Goodhead, 1994, 1999; Sutherland et al., 2000). The real effect of HZE on microorganisms is still unknown since during the previous space exposure experiments microorganisms were exposed and survived to non-ionizing parameters only (Rabbow et al., 2012, 2015).

In that contest, the STARLIFE-irradiation campaign (2013–2015) aimed to study the responses of astrobiological model microorganisms to increased doses of ionizing radiation and heavy ions, mimicking representatives of the GCR, never tested before. The dose rates used in the experiments were higher than those of natural GCR (Moeller et al., 2017).

Our model organism was the Antarctic cryptoendolithic black fungus *Cryomyces antarcticus* CCFEE 515, previously selected for astrobiological experiments for assessing the habitability of Mars, the likelihood of Lithopanspermia, i.e., the possible transfer of life via meteorites (Mileikowsky et al., 2000; Clark, 2001; Nicholson, 2009), and for the astronauts and planetary space protection (Hawrylewicz et al., 1962; Rummel, 2001; Nicholson et al., 2009; Horneck et al., 2010). In a space experiment the fungus has already survived 18 months real space exposition and Martian simulated conditions (Onofri et al., 2012, 2015).

*Cryomyces antarcticus* was isolated from the McMurdo Dry Valleys in Antarctica, considered as Mars analog due to cold temperatures, dryness and high UV irradiation (Selbmann et al., 2005; Onofri et al., 2012).

In the frame of STARLIFE experiments *C. antarcticus* has already shown a high resistance to gamma radiation up to 55.61 kGy (Co^60^) in dried condition (Pacelli et al., 2017b).

In a separate experiment the fungus has also demonstrated to tolerate densely (deuterons, ^2^H up 1,500 Gy) and sparsely (X-rays up to 300 Gy) ionizing radiation in physiological condition (Pacelli et al., 2017a). In this experiment *C. antarcticus* has been tested, with other astrobiological models, with accelerated Helium (150 MeV/nucleon) up to 1 kGy, as part of space-relevant ionizing radiations tested in the STARLIFE irradiation campaign (Moeller et al., 2017). Alpha particles are able to originate a high LET and a consequent higher direct biological effect (Cordero, 2017). The fungal response, after exposure in dried condition, was assessed by (i) cultivation test (CFU number), (ii) membrane damage assessment (PMA-qPCR), (iii) metabolic activity (XTT assay), (iv) DNA integrity (single gene PCRs and fingerprinting analysis), and (v) ultrastructural damage (TEM analysis).

## Materials and Methods

### Samples Preparation and Exposure Conditions

*Cryomyces antarcticus* CCFEE 515, an Antarctic cryptoendolithic black yeast-like micro-colonial fungus, was isolated from sandstone rock collected at Linnaeus Terrace (McMurdo Dry Valleys, Southern Victoria Land) by S. Onofri during the Antarctic expedition 1980-81. Samples were prepared as follows: cell suspensions (1,000 CFU) were spread on Petri dishes of MEA medium (malt extract, powdered 30 g/L; peptone 5 g/L; agar 15 g/L; Applichem, GmbH). Fungal colonies were incubated at 15°C for 3 months and dried under laminar flow in a sterile cabinet. Colonies were irradiated with accelerated helium ion (150 MeV/n, LET 2.2 keV/μm) doses (ranging from 50 to 1,000 Gy), at the HIMAC facility at the NIRS in Japan: the doses were 50, 100, 500, and 1,000 Gy. Controls (0 Gy) were kept in the lab at room temperature.

Dose rates used in this study were far beyond those that reach Mars’ surface and objects in outer space to simulate doses that organisms would receive over extended periods of time (Moeller et al., 2017; Verseux et al., 2017). All tests were performed in triplicate.

### Survival Assessment

#### Cultivation Test

Microorganism survival was determined by measuring colony forming ability as percentages of CFU. Three of the treated colonies were re-hydrated for 72 h in 1 mL of physiological solution (NaCl 0.9%); 0.1 mL (1,000 cells/mL) of the suspension was spread on Petri dishes supplemented with MEA (five replicates). Dishes were incubated at 15°C for 3 months and developing colonies were counted. Means and standard deviations were calculated. Statistical analyses were performed by one-way analysis of variance (ANOVA) and pair wise multiple comparison procedure (Tukey test, Box et al., 1978), carried out using the statistical software SigmaStat 2.0 (Jandel, United States).

#### Membrane Damage Assessment

Quantitative PCR after treatment with PMA was performed to assess the membrane integrity for the all irradiation doses.

After 3 days of re-hydration, PMA (Biotium, Hayward, CA, United States) at a final concentration of 200 μM was added to colonies for 1 h. PMA penetrates only damaged membrane cells, crosslinks to DNA after light exposure and thereby prevents PCR. DNA extraction, purification and quantitative PCR, used to quantify the number of fungal Internal Transcribed Spacer (ITS) ribosomal DNA fragments present in both PMA treated and non-treated samples, were performed according to Onofri et al. (2012). Before qPCR, DNAs were quantified and normalized at same concentration (2 ng/mL) using Qubit dsDNA HS Assay Kit (Life Technologies, United States). All tests were performed in triplicate.

#### Determination of Metabolic Activity by XTT Assay

Colorimetric assay of cellular viability, namely XTT assay, was performed according to the protocol in Kuhn et al. (2003). The XTT is converted to a colored formazan in the presence of cell metabolic activity. After irradiation, colonies of *C. antarcticus* were re-hydrated in 1 mL of Malt Extract (30 gr/L). After 10 days, fungal cells were washed, suspended in phosphate-buffered saline (PBS) and placed into 96 well plates, three wells for each condition. The XTT assay was performed adding 54 μL XTT (10 mg/ml)/menadione (2 mM) to each well. Plates were covered with foil and incubated, in agitation, at room temperature (24°C). Formazan product in the supernatant was detected by measuring the optical density at 492 nm (Labsystem Multiskan, Franklin, MA, United States) after 2, 3, 4, and 12 h of incubation.

### DNA Integrity Assessment

#### DNA Extraction, Single Gene PCR Reactions, and RAPD Analysis

DNA was extracted from colonies using Nucleospin Plant kit (Macherey-Nagel, Düren, Germany) following the protocol optimized for fungi (Selbmann et al., 2014).

Internal Transcribed Spacer and Small Subunit region (SSU) were amplified using BioMix (BioLine GmbH, Luckenwalde, Germany) adding 5 pmol of each primer and 2 ng of template DNA at final volume of 25 μL. The amplification was carried out using MyCycler Thermal Cycler (Bio-Rad Laboratories GmbH, Munich, Germany) equipped with a heated lid. The fungal rDNA regions were amplified using ITS4, ITS5, LR5, and LR7 and amplification conditions are as reported in Selbmann et al. (2011). Band intensities were measured and compared by using Image J software (Schneider et al., 2012). The whole genome was analyzed through fingerprinting analysis (Random Amplified Polymorphic DNA, RAPD protocol) and was performed using GGA_7_ primer according to Selbmann et al. (2011).

Primers specification as follows: ITS4 (TCCTCCGCTTATTGATATGC, White et al., 1990); ITS5 (GGAAGTAAAAGTCGTAACAAGG, White et al., 1990); LR5 (TCCTGAGGGAAACTTCG, Vilgalys and Hester, 1990); LR7 (TACTACCACCAAGATCT, Vilgalys and Hester, 1990); (GGA)_7_ (GGA GGA GGA GGA GGA GGA GGA, Kong et al., 2000).

### Ultrastructural Damage Assessment

After re-hydration, controls and colonies irradiated with medium and maximum irradiation dose (500 and 1,000 Gy) were prepared for TEM.

Colonies were treated with 5% glutaraldehyde/cacodylate sucrose buffer 0.1 M (pH 7.2) for 12 h at 4°C, washed three times in the same buffer for 1 h each at 4°C and fixed with 1% OsO_4_ + 0.15% ruthenium red in 0.1 M cacodylate buffer (pH 7.2) for 3 h at 4°C. Samples were washed in distilled water (two times for 30 min at 4°C), treated with 1% uranyl acetate in distilled water for 1 h at 4°C and washed in distilled water (two times, 30 min at 4°C). Samples were then dehydrated in ethanol solutions: 30, 50, 70% (15 min each, at room temperature) and 100% ethanol (1 h at room temperature); then, they were infiltrated in mixtures of ethanol 100%: LR White resin (Agar Scientific) (2:1 for 3 h; 1:1 for 3 h, 1:2 overnight), in rotator, at 4°C. The final step of the infiltration process was performed in pure resin for 36 h. Samples were then embedded in pure resin in gelatine capsules at 48–52°C for 2 days and blocks were cut by a Reichert-Jung E Ultracut ultramicrotome equipped with diamond knife. Ultrathin sections (60–80 nm) were collected on copper grids and stained with uranyl acetate and lead citrate; then they were observed with JEOL 1200 EX II Transmission Electron Microscope. The images have been acquired using a Veleta CCD camera (Olympus Soft Imaging Solutions).

## Results

### Survival Assessment

#### Cultivation Test

**FIGURE 1:**
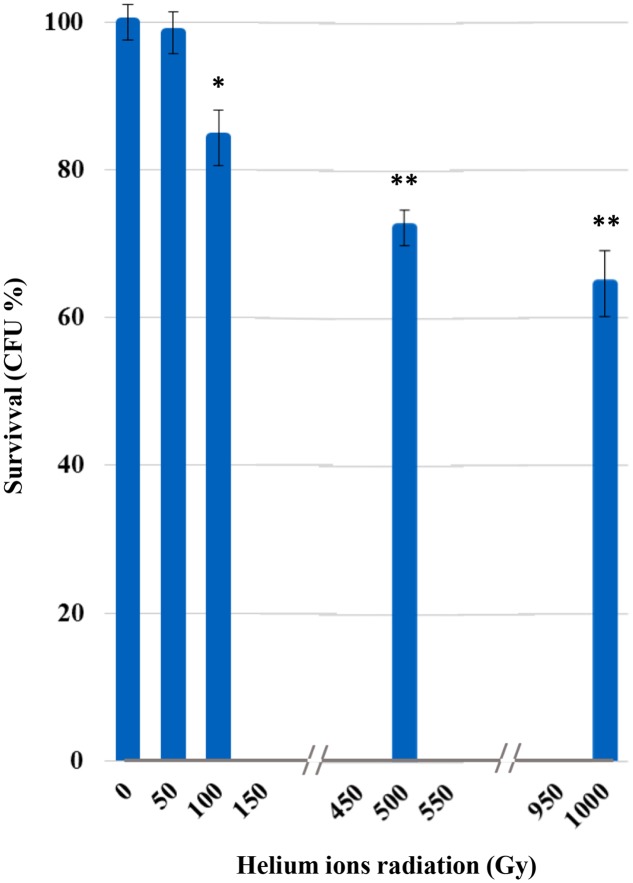
Cultivation test after helium ions radiation: percentages of *Cryomyces antarcticus* CFU after 50, 100, 500, and 1,000 Gy. Data were normalized with the control. Significant differences were calculated by Tukey test with ^∗^*p* > 0.05; and ^∗∗^*p* > 0.001.

Survival was not affected after irradiation at 50 Gy (**Figure 1**). From 100 Gy onward, a progressive increase of mortality was recorded with the increasing of irradiation doses; yet, *C. antarcticus* retained the colony-forming ability after all treatments and 65% of vitality with the respect to the laboratory control was still maintained at the highest dose applied (1,000 Gy).

#### Metabolic Activity

**FIGURE 2:**
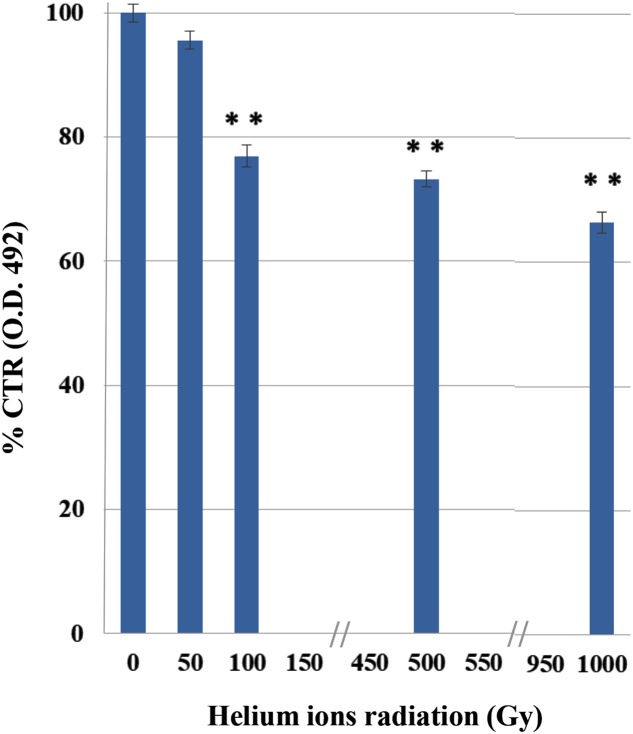
Effects of irradiation on metabolic activity of *C. antarcticus* cells by XTT assay after helium ions radiation. O.D. 492 nm, optical density at 492 nm. Data were normalized with the control. Significant differences were calculated by Tukey test with ^∗∗^*p* > 0.001.

The XTT results demonstrated a decrease in *C. antarcticus* metabolic activity, with the increasing of helium ions irradiation doses (**Figure 2**). This substance is cleaved to formazan by the succinate dehydrogenase system of the mitochondrial respiratory chain. Only living cells, possessing an intact mitochondrial membrane and also an intact cell membrane, do have active dehydrogenase. The first significant reduction was measured at 100 Gy (77%). However, 66% of metabolic activity compared to the laboratory control is still maintained at the highest doses, confirming the cultivation tests results and showing a good preservation of cell machinery after treatments.

#### Membrane Damage Assessment

The integrity of plasma membranes in irradiated cells was assessed by using qPCR after treatments with PMA; this molecule penetrates cells with compromised plasma membranes and inhibit DNA amplification.

**FIGURE 3:**
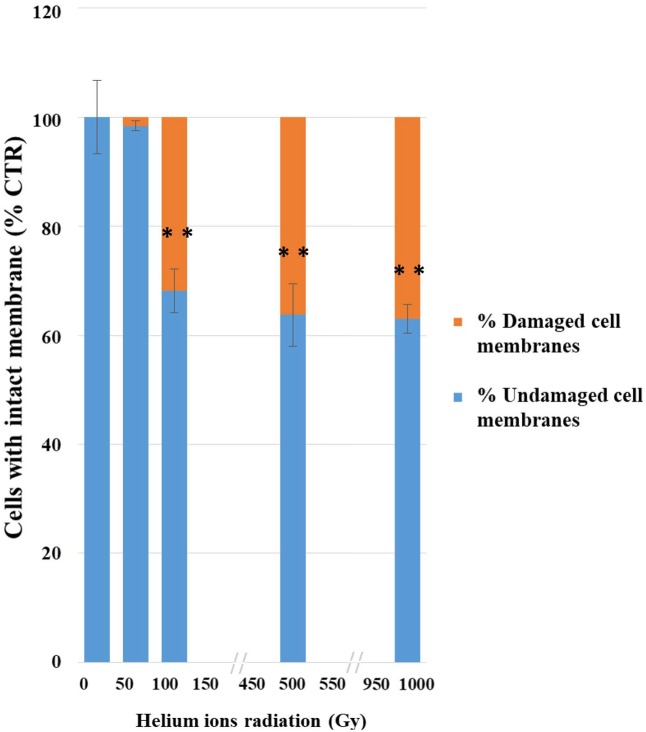
Results of PMA assay coupled with qPCR after helium ions radiation: 50, 100, 500, and 1,000 Gy. The graph shows percentages of *C. antarcticus* cells with intact membrane, normalized with the control. Percentages of *C. antarcticus* cells with damaged membrane (Red), and corresponding percentages of cells (Blue) with intact membrane. Significant differences were calculated by Tukey test with ^∗∗^*p* > 0.001.

This analysis also revealed a progressive damage with the increasing of treatments (**Figure 3**); no significant damage was present after the lowest treatment (50 Gy) and 68% damaged cells were recorded at 100 Gy. Besides up to 63% of cells still maintained membrane integrity after the highest dose (1,000 Gy).

### DNA Integrity Assessment

**FIGURE 4:**
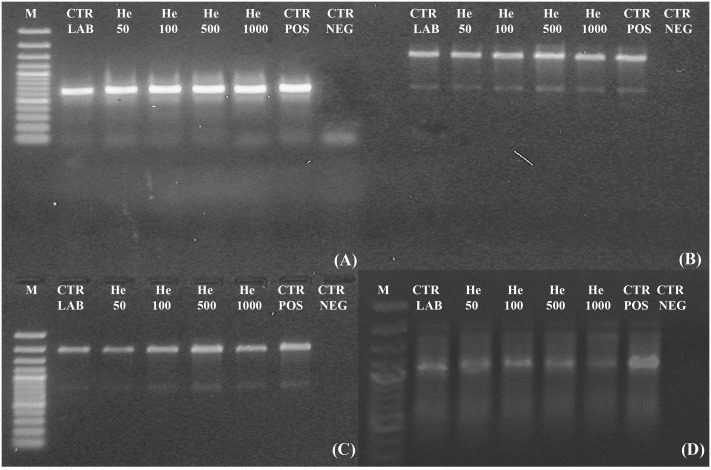
Assessment of the DNA integrity on *C. antarcticus* single gene PCR **(A)** 700 bp; **(B)** 1,600 bp; **(C)** 2,000 bp and **(D)** genomic DNA damage as revealed by RAPD assay. **M**: DNA ladder, **POS CTR**: fresh colonies, **NEG CTR**: PCR negative control.

Amplicons were obtained both for ITS and for ITS-LSU regions, fragments of 700, 1,600, and 2,000 bp of *C. antarcticus* DNA respectively, after irradiation treatments (**Figures 4A–C**). All bands were well-preserved in 700, 1,600, and 2,000 bp gene length; the gel analyses, performed with ImageJ Software, for all of them measured 100% relative density of the band till the highest irradiation dose. The RAPD profiles were preserved in all the conditions tested (**Figure 4D**). A reduction of band intensity in 1 kGy irradiated samples was visible for the band round 1,000 bp while the highest molecular weight (MW) bands were maintained. This is difficult to explain since the highest MW bands (about 2,200 bp) of the RAPD profiles would have disappeared first in case of DNA damage (Atienzar et al., 2002); moreover, in the single gene amplification, band intensity was maintained even for DNA amplicons of 2,000 bp. In the overall, results indicate that these kinds of treatments did not cause any detectable damage to the fungal DNA, at least with the techniques here used; unfortunately, for organisms with such a thick cell wall other procedures, theoretically more appropriate to reveal DNA damage as the comet assay, are not applicable.

The possible mutational burden gained by the fungus after irradiation was studied by amplicon sequencing. The electropherograms appeared perfectly preserved with no single mutation detectable (data not shown).

### Ultrastructural Damage Assessment

**FIGURE 5:**
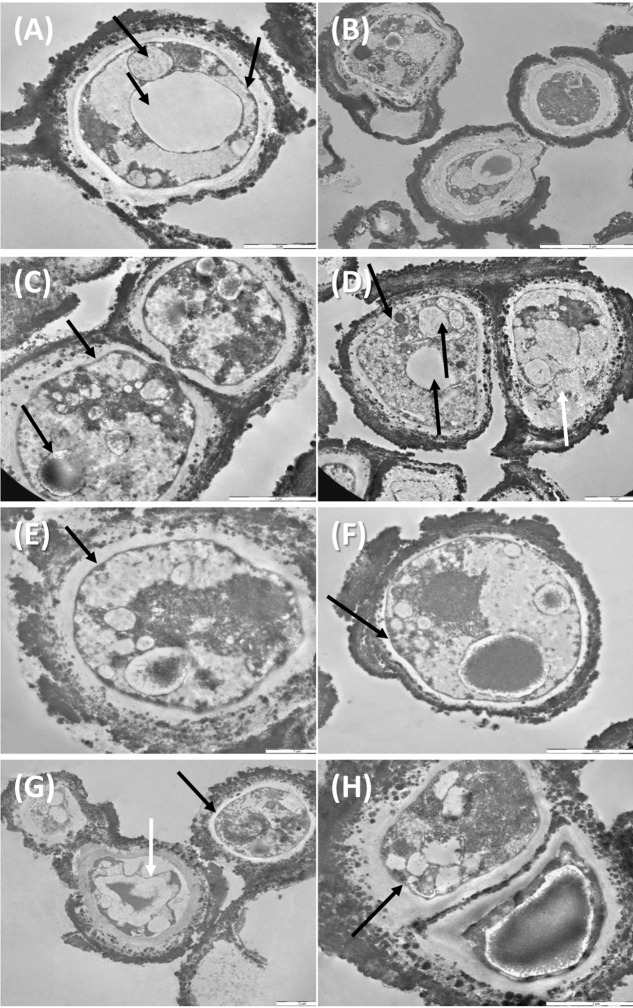
TEM observations of *C. antarcticus*: Control **(A**,**B)**; 500 Gy treatments, **(C**–**F)** cell membrane and organelles are perfectly preserved (black arrows, **C–F**), damaged membrane (white arrow, **D**); 1,000 Gy treatments **(G**,**H)** plasmolysis and amorphous cytoplasm (white arrow, **G**) well-preserved membrane (black arrow, **G,H**).

Despite the dehydration treatments, control cells were perfectly preserved (**Figures 5A,B**), with well-organized cytoplasm and cell membrane integrity (**Figure 5A**, right black arrow). Nucleus and vacuole (**Figure 5A**, black arrow) were well-visible.

Even samples treated with 500 Gy were rather well-preserved (**Figures 5C–F**). Cell membranes were always maintained (**Figures 5C–F**, black arrows) except for cell on the right, **Figure 5D**, white arrow, where also cytoplasm organization was lost; organelles as nuclei, vacuoles and lipid bodies were also visible in most of the cells (**Figures 5C,D**, black arrow).

Membrane integrity and cytoplasm organization were maintained also at the highest dose of 1,000 Gy (**Figures 5G,H** black arrows), while plasmolysis and amorphous cytoplasm are evident in the cell documented in **Figure 5G**, white arrow. These results support data recorded with cultivation test, proving that a very good vitality was maintained even at the highest dose applied.

## Discussion

Given past and present interest of the Space Agencies in the long-term effects of ionizing radiation on survival, it is surprising that relatively few studies have been addressed to this issue. Helium ions inducing a high-linear energy transfer (LET) are of special interest for their potential biological action. Efforts have been played not only to elucidating fundamental radiobiological mechanisms, but also to applied aspects such as radiation therapy and human exposure to radiation during space flight. In this study we investigated the responses of a eukaryotic model, the fungus *C. antarcticus*, after irradiation with accelerated helium (high-energy particles), as space-relevant radiation. Helium nuclei are densely ionizing radiation sources (high LET radiation) and are of special concern as major source of radiation exposure in outer space (Horneck, 1994). In particular, ions of cosmic radiation are used to set the ultimate limit on (micro-)organisms survival in space (Horneck et al., 1994).

HZE particles (heavy ions) are the biologically most effective component of GCR; doses applied in this experiment are very high even compared to real space, where a putative organism should be exposed over extended periods of time to receive a comparable dose. One kGy of helium radiation corresponds to an average of approximately 2.0 × 10^5^ He ions (Verseux et al., 2017). As measured during several space missions, for instance during the LDEF mission (Horneck et al., 1994), fluence was very low, around 6 × 10^-5^ particles/year-μm^2^; as a consequence, they would not represent the major danger for a putative microorganism in space (Horneck et al., 2010).

Despite their high potential danger, alpha particles are more easily screened that other harmful radiation as beta or, even more, gamma radiation. The very thick and strongly melanized cell wall of our model organism may have represented an effective barrier for stopping He nuclei, even associated to high LET. In fact, no reduction of survival, metabolic activity or even cell with damaged membrane was detected at 50 Gy. Survival, PMA, and XTT tests gave a progressive reduction at the other irradiation doses. We assume that the first dose was not that high for our test organism to cause appreciable damage. The effect of treatments on the cells was progressively higher, and comparable among different tests, at 100, 500, and 1,000 Gy.

This may justify the high survival and metabolic activity observed even at the maximum dose tested in the experiment where still 64% of living cells and 66% of metabolic activity were maintained, respectively.

Accordingly, still 63% of cells with intact membrane were present after PMA-qPCR assay at the highest irradiation dose (1,000 Gy). A general good membrane integrity was also observed with TEM; cell membrane was mostly preserved even at high doses applied while damages were visible in some cells at 1,000 Gy treatment in terms of membrane integrity and cytoplasm preservation (**Figure 5**). As reviewed by Horneck et al. (1994), DNA double-strand breaks (DSBs) are the most severe type of damage induced by HZE particles in microorganisms, as determined in cells of *Escherichia coli*, *Deinococcus radiodurans*, and *Bacillus subtilis*. Nevertheless, DNA damage was not detectable at all in our model *C. antarcticus*; single gene amplifications were successful even at highest dose and longest gene sequences tested; accordingly, the fingerprinting profiles were perfectly maintained (**Figure 4**).

These results were consistent with the other STARLIFE models as the cyanobacterium *Chroococcidiopsis* sp. and the lichen *Circinaria gyrosa* that also survived the treatment (de la Torre et al., 2017; Verseux et al., 2017). *C. antarcticus* has been extensively tested for its resistance to the space-relevant radiation (Onofri et al., 2012, 2015; Pacelli et al., 2016, 2017b). However, the tolerance mechanisms of this astrobiological model to outer space environmental stresses are still poorly understood but they could be related to its ability to survive desiccation. In fact, during anhydrobiosis, a state of metabolic inactivity after desiccation (Kranner et al., 2008), radiolysis of intracellular water, that is the most significant source of hazardous reactive oxygen species (Kovács and Keresztes, 2002), does not occur or is almost absent.

This radio-resistance of cells in a dry state was previously observed in bacteria as *Deinococcus radiodurans* (Bauermeister et al., 2011) and *Halobacterium salinarum* (Leuko et al., 2015; Leuko and Rettberg, 2017; Verseux et al., 2017) and was also addressed to reduced number of reactive oxygen species, and lower rates of harmful chemical reactions, when cells are in a dry state (Bauermeister et al., 2011).

In our study, the fungal DNA remained perfectly detectable even after exposure to 1 kGy of He^2+^ ions. These results are consistent with what previously observed when the fungus was treated with gamma-ionizing radiation (Pacelli et al., 2017b) and to other factors encountered in space and under Mars-like conditions (Pacelli et al., 2016). The DNA detectability after radiation treatments was reported for other microorganisms tested in the frame of STARLIFE project (Verseux et al., 2017). Those findings are relevant to search-for life missions on Mars, confirming this molecule as putative biosignature since its presence provides unequivocal proof of present or recently past life even if, it must be taken into account, that extracellular DNA may be bound to minerals or destroyed once hydrated (Aerts et al., 2014; Mojarro et al., 2017). Life detection missions to Mars focus on detecting biomarkers as evidence for extant or extinct life (Parnell et al., 2007; De Vera et al., 2012). The surface of Mars is continuously exposed to high levels of cosmic radiation, which could be deleterious for both microorganisms survival and persistence of molecular biosignatures such as DNA (Dartnell et al., 2007). Being amplified even after helium ions and ionizing radiation (Pacelli et al., 2017b; Verseux et al., 2017), we largely demonstrated that DNA has a high intrinsic stability and that it could be account as biosignature.

Even if space radiation is quite more complex and cannot be fully simulated on Earth, these results may help us to better understand the effects of helium ions radiation in future astrobiological simulation and real exposure experiments on fungal models and eukaryotic cells in general. This approach will more precisely elucidate the effects of space radiation on fungal resistance and aid in developing personalized radiological countermeasures for astronauts in the frame of planetary protection.

## Author Contributions

CP, SO, and LS designed the study. CP, RM, and AF performed the experiment. CP, SO, LS, and LZ analyzed and interpreted the data. CP wrote a first draft of the manuscript, which was corrected, revised and approved by all authors.

## Conflict of Interest Statement

The authors declare that the research was conducted in the absence of any commercial or financial relationships that could be construed as a potential conflict of interest.

## Abbreviations

CFU: colony-forming units
GCR: galactic cosmic radiation
HIMAC: Heavy Ion Medical Accelerator in Chiba
HZE: high atomic charge and energy
LET: linear energy transfer
MEA: malt extract agar
NIRS: National Institute of Radiological Sciences
PCR: polymerase chain reaction
PMA-qPCR: Propidium MonoAzide-quantitative polymerase chain reaction
RAPD: random amplified polymorphic DNA
SPE: solar energetic particle events
TEM: transmission electron microscopy
XTT: colorimetric assay of cellular viability with 2,3-*bis*(2-methoxy-4-nitro-5-sulfophenyl)-5- [(phenylamino) carbonyl]-2H-tetrazolium hydroxide
